# Lipid analysis in an aging population

**DOI:** 10.18632/aging.101811

**Published:** 2019-02-07

**Authors:** Vincent A. Pallazola, Vasanth Sathiyakumar, Seth S. Martin

**Affiliations:** 1 Johns Hopkins Ciccarone Center for the Prevention of Cardiovascular Disease, Baltimore, MD 21287, USA

**Keywords:** low density lipoprotein cholesterol, triglycerides, Martin-Hopkins LDL-C equation, personalized medicine, aging

Due to an aging U.S. population, the incidence of cardiovascular disease is rapidly increasing, with an expected rise in coronary heart disease prevalence by 43% (increase by 5 million) and an associated cost of $70 million by the year 2030 [1]. The Pooled Cohort Equations, which estimate 10-year atherosclerotic cardiovascular disease (ASCVD) risk and are fundamental to current statin, aspirin, and blood pressure prevention guidelines, are validated for patients up to 75 years old and weighted heavily by age. Among modifiable risk factors, cholesterol reduction with statins and adjunctive therapies is a major point of intervention for cardiovascular prevention in the elderly. Accurate low density lipoprotein cholesterol (LDL-C) calculation is important for this population, as discriminate cholesterol reduction has the potential to provide substantial absolute benefit in a select subset of patients [1].

The Friedewald equation [2], serving as the global standard for LDL-C calculation for several decades, was initially validated in the fasting state. The 2016 European Atherosclerosis joint consensus statement, however, has shifted to recommend non-fasting lipid testing given its convenience and accuracy at a population level [3]. Non-fasting lipid assessment also helps avoid hypoglycemic events with fasting, to which elderly patients may be more prone.

Acknowledgement of the Friedewald equation’s limitations at low LDL-C, high triglycerides (TG), and the non-fasting state has prompted significant efforts to improve upon the equation. The Friedewald equation is prone to error in the non-fasting state and diseases such as diabetes due to alteration in the ratio of TG to very low density lipoprotein cholesterol (VLDL-C) [4]. The comparatively low fixed factor set by the Friedewald equation tends to overestimate VLDL-C in these contexts, leading to underestimation in LDL-C and therefore potential for undertreatment. Metabolic syndrome, which increases in prevalence with age and is associated with severe hypertriglyceridemia, has a similar effect on Friedewald estimation via displacement of TG to non-VLDL particles.

Friedewald and colleagues recognized in their seminal publication that at lower LDL-C levels, small error in VLDL-C estimation might promote significant error in LDL-C estimation [2]; this was originally tolerated given that few individuals had low LDL-C and treatments such as statins, ezetimibe, and proprotein convertase subtilisin/kexin type 9 (PCSK9) inhibitors were not available. In our recent study, approximately 20% of individuals with Friedewald-estimated LDL-C <70 mg/dL were shown to have LDL-C ≥70 mg/dL [5], a key therapeutic cutoff used in several international guidelines for high-risk individuals. Limitations in accuracy with the Friedewald equation when applied in the modern era of clinical care prompted development and validation of a novel method for LDL-C estimation that improves accuracy in both fasting and non-fasting settings: the Martin-Hopkins LDL-C equation [6].

Sathiyakumar et al. (2018) performed a cross-sectional analysis of over 1.5 million patients from the second harvest of the Very Large Database of Lipids that demonstrated improvement in LDL-C accuracy with the new Martin-Hopkins method in non-fasting as well as fasting contexts [7]. Rapid ultracentrifugation was used to directly measure LDL-C, with accuracy defined as percentage of LDL-C measured by ultracentrifugation falling within an estimated Friedewald or Martin-Hopkins LDL-C category by clinical cut point (e.g., <70 mg/dL, 70-<100 mg/dL). In patients with low estimated LDL-C (<70 mg/dL), accuracy was furthermore assessed via stratification by triglyceride levels. In contrast to the Friedewald equation, LDL-C accuracy was largely preserved with the Martin-Hopkins equation regardless of fasting status, LDL-C range, or triglyceride level (up to 400 mg/dL). The greatest improvement in reclassification was demonstrated in non-fasting patients, particularly in cases of low LDL-C and high TG. Specifically, within the LDL-C <70 mg/dL cut point, use of the novel method in non-fasting patients improves misclassification rates of LDL-C by 20-29 or ≥30 mg/dL from 8.1% to 0.2% and from 3.6% to 0%, respectively.

These findings suggest that the Martin-Hopkins method of LDL-C estimation is more accurate in non-fasting than the Friedewald equation, and that fasting status has minimal effect on LDL-C classification with this novel method. Although previous gross assessment at the population level argues for the routine use of non-fasting samples on a population scale [8], Sathiyakumar et al. (2018) show that when patients are isolated at a lower LDL-C cut point (<70 mg/dL) and higher TGs, correlation between Friedewald estimated LDL-C and ultracentrifugation decreases and accuracy is considerably compromised by non-fasting status. With international guidelines focused on attaining lower and lower LDL-C levels and the development of new medications such as ezetimibe and PCSK9 inhibitors to treat to these levels, the need for accurate estimation at low LDL-C is much more important than it was when the Friedewald equation was developed.

Although large-scale clinical trials and cohort studies have disproportionately underrepresented the elderly population, this group tends to have the highest incidence of cardiovascular disease and may see some of the greatest benefit from lipid lowering therapies. In the elderly, accurate LDL-C calculation is essential as appropriately aggressive lipid treatment is likely to have significant absolute benefit on reducing ASCVD events, while at the same time over-treatment may have a significant negative impact given the heightened risk of medication adverse effects and polypharmacy. The enhanced accuracy of the Martin-Hopkins equation may help correctly isolate those who would benefit from more aggressive reduction of cholesterol to improve outcomes.

The Martin-Hopkins method arises from big data analytics, providing a precision medicine rather than one-size-fits-all approach to LDL-C calculation. It is being incorporated worldwide given its low-cost implementation and significant accuracy benefits. By using the Martin-Hopkins equation, estimation of LDL-C could be more reliably liberalized to a non-fasting approach, which would be convenient for patients, clinicians, and clinical laboratories.

## References

[1] Mortensen MB, Falk E. J Am Coll Cardiol. 2018; 71:85–94. 10.1016/j.jacc.2017.10.080 29301631

[2] Friedewald WT, et al. Clin Chem. 1972; 18:499–502. 4337382

[3] Catapano AL, et al. Eur Heart J. 2016; 37:2999–3058. 10.1093/eurheartj/ehw272 27567407

[4] Kissebah AH, et al. J Clin Invest. 1983; 71:655–67. 10.1172/JCI110812 6338042 PMC436915

[5] Whelton SP, et al. J Clin Lipidol. 2017; 11:1065–72. 10.1016/j.jacl.2017.05.005 28826567

[6] Martin SS, et al. JAMA. 2013; 310:2061–68. 10.1001/jama.2013.280532 24240933 PMC4226221

[7] Sathiyakumar V, et al. Circulation. 2018; 137:10–19. 10.1161/CIRCULATIONAHA.117.030677 29038168

[8] Langsted A, et al. Circulation. 2008; 118:2047–56. 10.1161/CIRCULATIONAHA.108.804146 18955664

